# Do patients with bipolar disorders have an increased risk of stroke? A systematic review and meta-analysis

**DOI:** 10.12669/pjms.40.11.10732

**Published:** 2024-12

**Authors:** Lingli Guo, Fanghua Wu, Jiaojiao Yang

**Affiliations:** 1 Lingli Guo, Department of Psychosomatic Disorders, Huzhou Third Municipal Hospital, The Affiliated Hospital of Huzhou University, Huzhou, Zhejiang Province 313000, P.R. China; 2 Fanghua Wu, Department of Psychosomatic Disorders, Huzhou Third Municipal Hospital, The Affiliated Hospital of Huzhou University, Huzhou, Zhejiang Province 313000, P.R. China; 3 Jiaojiao Yang, Department of Psychosomatic Disorders, Huzhou Third Municipal Hospital, The Affiliated Hospital of Huzhou University, Huzhou, Zhejiang Province 313000, P.R. China

**Keywords:** Stroke, Mental disorders, Thromboembolism, Risk, Bipolar dirorder

## Abstract

**Objective:**

The objective of the current review aimed to assess if patients with bipolar disorder (BD) have an increased risk of stroke.

**Methods:**

A literature search was conducted on PubMed, Embase, ScienceDirect, and Web of Science for studies published from inception to 20^th^ June 2023 and reporting the association between BD and stroke.

**Results:**

Eight studies with 9187894 participants were eligible. Meta-analysis of all eight studies demonstrated that BD patients have an increased risk of stroke as compared to non-BD individuals (HR: 1.60 95% CI: 1.24, 2.05 I^2^=94%). Results did not change in significance on sensitivity analysis. However, results varied on subgroup analysis based on study location, study type, sample size, and follow-up duration. The effect magnitude was unchanged based on the adjustment of diabetes and hypertension and study quality.

**Conclusion:**

Patients with bipolar disorder (BD) may have an increased risk of stroke in comparison to the general population. The results are limited by the retrospective nature of the data and high inter-study heterogeneity which demand caution in the interpretation of the outcomes.

***Protocol Registration:*** PROSPERO (CRD42023434365), https://www.crd.york.ac.uk/prospero/

## INTRODUCTION

Bipolar disorder is a recurrent chronic mental disorder affecting a significant number of patients worldwide.^1^ Approximately 1% of the global population is affected by BD without any differences based on country of origin, ethnicity, or socioeconomic status.^2^ BD is a major cause of functional and cognitive decline in young adults with subsequent disability increasing mortality rates, particularly by suicide.^3^ Patients with BD have a two-fold increased risk of mortality as compared to the general population.^4^,^5^ The mortality rates have been attributed to the higher risk of coronary artery disease, cerebrovascular disease, heart failure, and hypertension in patients with BD.^6^

Stroke is one of the most common cerebrovascular diseases leading to the second-highest number of deaths around the world^7^ and the most in China.^8^ It remains the second most common cause of disability around the world contributing to a major healthcare economic burden on society.^9^ The high incidence of stroke has prompted researchers to look for risk factors for the disease and incorporate preventive strategies in public healthcare programs.^10^

Recent research has shown that the prevalence of diabetes and metabolic syndrome with its entire spectrum of obesity, dyslipidemia, and hypertension is higher in BD patients as compared to the general population.^11^ Research has also shown that BD patients have increased risk of thromboembolism.^12^ Diabetes and metabolic syndrome are well-recognized risk factors of stroke.^13^ Any thromboembolic episode can lead to increased risk of stroke.^10^ Considering these numerous risk factors of stroke in BD patients, several studies have examined if BD patients have a higher risk of stroke as compared to non-BD individuals.

Several studies have examined the risk of stroke in BD but results have varied and there is no consensus.^14^–^16^ Previously, Prieto et al^17^ and Yuan et al^18^ in their meta-analysis studies had examined the association between BD and stroke and both have noted higher risk of stroke in BD patients. However, these review could include just three and seven studies respectively. Moreover, the review of Yuan et al^18^ had a major limitation wherein three studies using overlapping data from the same healthcare database were included. To overcome these limitations and improve quality of evidence, we conducted an updated literature search, incorporated new studies, and deleted overlapping data to quantitatively assess if BD leads to an increased risk of stroke.

## METHODS

The eligibility criteria for inclusion in the review were Cohort or case-control studies which were to examine the association between BD and subsequent risk of stroke. Moreover, studies were to report the adjusted effect size of the relationship between BD and stroke.

### Inclusion & Exclusion Criteria:

Studies on other mental illnesses and not reporting separate data on BD were excluded. Similarly, studies reporting thromboembolic disorders as outcomes but not separate data on stroke were also not eligible. If two studies used the same database with a similar inclusion period, the studies with the maximum sample size were included. Additionally, cross-sectional studies, editorials, studies in non-English language, and non-peer-reviewed studies were excluded.

### Search source and strategy:

A literature search was conducted on PubMed, Embase, ScienceDirect, and Web of Science by two reviewers independently (LG & FW). The last date of the search was 20^th^ June 2023. Keywords used were “bipolar disorder”, “bipolar depression”, “manic disorder”, “bipolar affective disorder”, “stoke”, “cerebrovascular disease”, “intracerebral hemorrhage”, AND “thromboembolism”. Using “AND” and “OR” various search strings were generated to optimize the search. We also searched the reference lists of included studies for any missed articles. Google Scholar was used to search gray literature.

Two reviewers scrutinized the results of all databases independently (LG & FW). First, the searched articles were collected and all duplicates were removed. The reviewers then scrutinized every original article by studying their titles and abstracts. Articles not in the context of the review were excluded and the rest were selected for full-text analysis. All discords between reviewers were solved by discussion with the third reviewer (JY).

### Extracted data and outcomes:

Two reviewers (FW & JY) sourced data required for the review using a pre-formatted table name of the first author, year of publication, country, database of the study, sample size, number of stroke patients, age and gender details, method of identification of BD and stroke, details of control group, adjusted covariates, follow-up, and outcome data were extracted by each reviewer independently. Outcome was the risk of stroke.

### Risk of bias analysis:

All included studies were examined for bias using the Newcastle Ottawa Scale (NOS).^19^ The NOS has three components: representativeness of the study cohort, comparability, and measurement of outcomes. The components have four, two and three points respectively with a maximum of nine points.

### Statistical analysis:

Reporting of the review was based on the PRISMA guidelines.^20^ The software used for the meta-analysis was “Review Manager” (RevMan, version 5.3). Individual study outcomes were sourced and pooled to generate the total hazard ratio (HR) with 95% confidence intervals (CI). The random-effects model was used. Subgroup analysis was done based on the location of the study, study type, sample size, follow-up, adjustment for diabetes and hypertension, NOS score, diagnosis of BD, and publication year. Publication bias was checked with funnel plots. The I^2^ and Q statistics were the tools to assess inter-study heterogeneity. Outliners in the meta-analysis were checked during the sensitivity analysis.

## RESULTS

**Fig.1:**
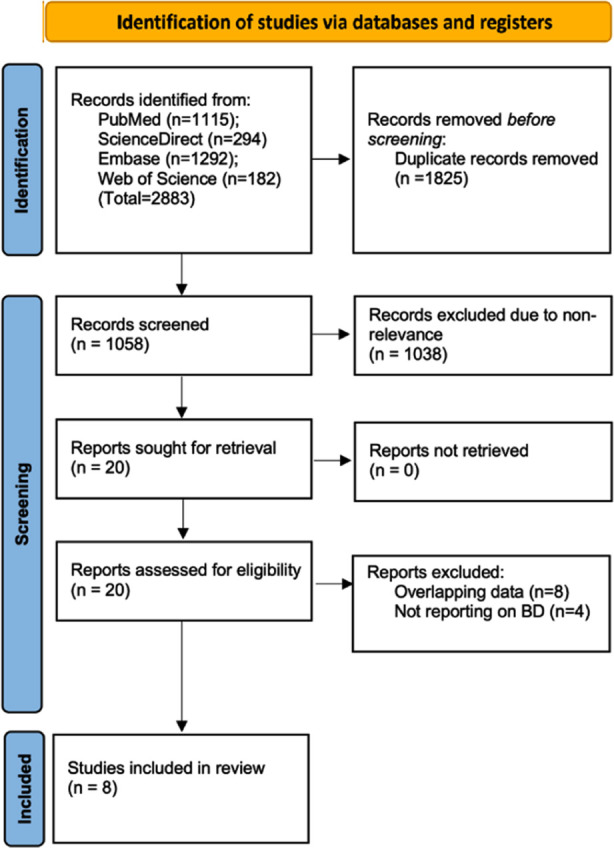
PRISMA study flowchart demonstrating search results at each stage.

The total number of studies from all databases was 2883. Twelve studies were excluded and the remaining eight were assessed in the review^5^,^14^,^15^,^19^-^23^ (Fig.1).

### Baseline characteristics of studies:

The extracted data from the studies are shown in Table-I. The eligible studies were conducted in Denmark, Taiwan, Scotland, Sweden, the USA, Lebanon, and Korea. The studies were published between 2004 to 2023 and included more than 9187894 participants. In one study^23^ the overall sample size was not clearly reported. Two studies were case-control and the rest were cohort. The follow-up of studies varied from 7 to 15.8 years. The quality of most studies was good with an NOS score of eight or nine. Only one study had a NOS score of seven. (Supplementary Table-I).

**Table-I:** Baseline data extracted from the included studies.

Study	Database	Location	Study type	Sample size	Age (year)	Male gender (%)	Identification of BD	Identification of stroke	Number of patients with stroke	Control population	Covariates adjusted	Follow-up (years)	NOS score
Nilsson 2004^14^	Danish National Hospital Register	Denmark	CC	83387	58.6	40	ICD codes	ICD codes	1572	Diagnosis of osteoarthritis	Gender, age and alcohol/drug abuse	17	6
Laursen 2011^21^	Danish Central Psychiatric case register	Denmark	Cohort	2450812	15-52	NR	ICD codes	ICD codes	NR	Individuals with no psychiatric diagnosis	Sex, age and calendar time	12	8
Wu 2013^22^	National Health Insurance Research Database	Taiwan	Cohort	84105	43.2	45.8	ICD codes	ICD codes	1915	Individuals without BD matched for age and sex	Sex, age, hypertension, diabetes, hyperlipidemia, level of urbanization, enrollee category, patient, physician and hospital variables	7	9
Westman 2013^15^	Swedish Total Population Register	Sweden	Cohort	10631	NR	NR	ICD codes	ICD codes	450	Individuals without BD and schizophrenia	Sex, calendar year and age	15.8	9
Prieto 2016^16^	Rochester Epidemiology Project	USA	Cohort	668	37	48	Clinical diagnosis	ICD codes	91	Same sex matched individual free of myocardial infarction, stroke and BD	Alcoholism, hypertension, diabetes, and smoking	16	8
Jackson 2020^23^	Scottish population-based records	Scotland	Cohort	NA	NA	NA	ICD codes	ICD codes	2600	Individuals with no psychiatric diagnosis	History of a psychiatric disorder, age, gender, time period and area-based deprivation index	14	8
Maalouf 2023^25^	Hospitals in Beirut and Mount Lebanon	Lebanon	CC	564	64	45	Mood disorder questionnaire	Computed tomography or Magnetic resonance based diagnosis	113	Gender matched individuals without BD and stroke	Age, marital status, educational level, hypertension, dyslipidemia, diabetes, heart diseases, atrial fibrillation, asthma-COPD, obesity, Mediterranean diet adherence, physical activity, schizophrenia, alcohol use disorder	NR	7
Park 2023^24^	National Health Insurance Service	Korea	Cohort	6557727	30.9	59	ICD codes	ICD codes	10844	Individuals with no psychiatric diagnosis	Age, sex, hypertension, diabetes mellitus, dyslipidemia, metabolic syndrome, chronic kidney disease, current smoking, heavy alcohol consumption, regular physical activity, and low-income level	7.6	9

BD, bipolar disorder; CC, case-control; ICD, international classification of diseases; NR, not reported; NOS, Newcastle Ottawa scale.

**Supplementary Table-I:** Risk of bias analysis based on the Newcastle Ottawa (NOS) scale.

Study	Selection of sample	Comparability	Outcome assessment	NOS score
Nilsson 2004^14^	**	**	**	6
Laursen 2011^21^	****	**	**	8
Wu 2013^22^	****	**	***	9
Westman 2013^15^	****	**	***	9
Prieto 2016^16^	****	**	**	8
Jackson 2020^23^	****	**	**	8
Maalouf 2023^25^	****	**	*	7
Park 2023^24^	****	**	***	9

### Meta-analysis:

**Fig.2:**
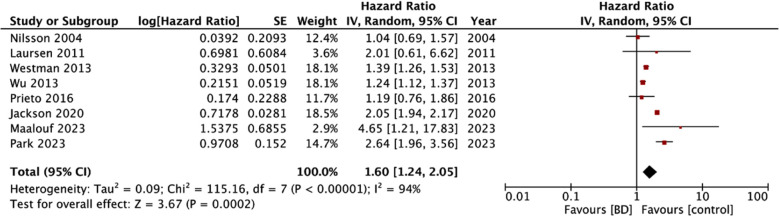
Meta-analysis of the association between BD and risk of stroke.

**Fig.3:**
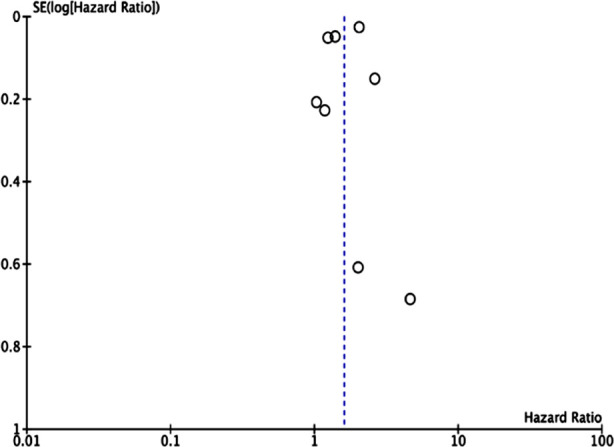
Funnel plot for assessing publication bias.

Meta-analysis of all eight studies demonstrated that BD patients have an increased risk of stroke as compared to non-BD individuals (HR: 1.60 95% CI: 1.24, 2.05 I^2^=94%) (Fig.2). There was no publication bias among the studies (Fig.3). We excluded one study at a time for sensitivity analysis and noted no change in the significance of the results.

### Subgroup analysis:

Details of various subgroup analyses are shown in Supplementary Table-II. Based on location, the single American study found no significant relation between BD and the risk of stroke. However, the results were significant for Asian and European studies. Based on study type, results were significant for cohort studies but not for case-control studies. Similarly, analysis of smaller sample-sized studies (<15000) generated significant results but not for larger sample-sized studies (>15000). Results were significant for studies with longer duration of follow-up (>10 years) but not for shorter follow-up (<10 years). Also, results were significant for studies using international classification of disease codes but not for studies using clinical diagnosis. Subgroup analysis for studies based on adjustment for diabetes and hypertension, NOS score, and publication year (pre and post-2015) did not change the results.

**Supplementary Table-II:** Subgroup analysis for the association between BD and risk of stroke based on different variables.

Variable	Groups	Studies	Hazard ratio [95% confidence intervals]
Location	Asian	3	2.09 [1.05, 4.19]
	American	1	1.19 [0.76, 1.86]
	European	4	1.53 [1.10, 2.12]
Study type	Cohort	6	1.64 [1.25, 2.15]
	Case control	2	1.91 [0.45, 8.07]
Sample size	<15000	3	1.42 [1.03, 1.95]
	>15000	4	1.56 [0.97, 2.50]
Follow-up	<10 years	2	1.79 [0.85, 3.74]
	>10 years	5	1.46 [1.08, 1.97]
Adjusted for diabetes and hypertension	Yes	4	1.76 [1.06, 2.92]
	No	4	1.53 [1.10, 2.12]
Newcastle Ottawa Score	9	3	1.57 [1.21, 2.05]
	<9	5	1.61 [1.05, 2.47]
Diagnosis of BD	ICD codes	6	1.60 [1.22, 2.10]
	Clinical	2	2.02 [0.55, 7.42]
Publication year	Pre-2015	4	1.30 [1.18, 1.44]
	Post-2015	4	2.04 [1.50, 2.76]

BD, bipolar disorders; ICD, international classification of diseases.

## DISCUSSION

This systematic review and meta-analysis of eight studies showed that patients with BD have increased risk of stroke as compared to those without BD. The results did not change on sensitivity analysis. However, the results became non-significant in several subgroup analyses.

Mental disorders contribute to a major chunk of the global disease burden leading to increased disability and mortality.^26^ Patients with psychiatric disorders including BD and major depression have a reduced life expectancy of up to 20 years.^27^,^28^ While suicide is one important factor contributing to the high mortality rates, a large proportion is also due to natural causes like cardiovascular and cerebrovascular diseases.^29^ Nevertheless, estimates on the elevated risk of cardiovascular and cerebrovascular disorders amongst BD patients have varied in literature. A population-based study from Taiwan on 70225 participants has shown an elevated risk of acute myocardial infarction amongst patients with BD as compared to individuals without any mental disorders.^30^ Contrarily, Prieto et al^16^ have shown that the elevated risk of both stroke and myocardial infarction in BD does not remain statistically significant after adjustment of cardiovascular risk factors like alcoholism, hypertension, diabetes, and smoking. To overcome the variability of outcomes in the literature, we performed an updated and detailed literature review of studies examining the association between BD and risk of stroke.

The results of our meta-analysis showed that patients with BD had a 60% higher risk of stroke as compared to the general population. Overall, we noted no outliner study in the meta-analysis as the results were statistically significant even after the sequential exclusion of individual studies. Our results concur with the previous review of Yuan et al^18^ wherein they reported a statistically significant increased risk of stroke amongst BD patients. However, three of the seven included studies in their review were from Taiwan using the same National Health Insurance database with overlapping study duration.^22^,^31^,^32^ Repetitive inclusion of the same sample demonstrating positive outcomes leads to bias in the meta-analysis and reduces the strength of the evidence.

This updated review excluded overlapping data and added three new studies from different countries thereby significantly increasing the power of the meta-analysis and improving the reliability of the results. By updating the review to include studies from other regions, the current analysis also expands the generalizability of outcomes and provides the best possible evidence on the relationship between BD and stroke in literature.

An important caveat in our analysis was the high inter-study heterogeneity which necessitates caution in the interpretation of results. Inter-study heterogeneity could be due to several factors like variations in the study population, study methodology, identification of BD and stroke patients, follow-up, etc. In this context, several subgroup analyses were conducted to assess the impact of these variables on the effect size. We noted non-significant results in multiple subgroup analyses of case-control studies, studies with <10 years of follow-up, studies using clinical diagnosis, and the single American study. A potential reason for the non-significant results could be the low number of studies in these subgroup analyses. Importantly, for the subgroup analyses based on adjustment of diabetes and hypertension and study quality, there was no change in the significance of the results. Both diabetes and hypertension are well-recognized risk factors for stroke causing an increased risk of atherosclerosis and subsequent thromboembolism.^33^ Adjustment of such important confounders is necessary to assess the true association between BD and the risk of stroke.

There could be several reasons for the high risk of stroke amongst BD patients. Patients with mental disorders often have poor lifestyles, substance abuse issues and receive inadequate treatment for medical conditions due to social deprivation all of which increase the risk of stroke.^34^ The use of psychotropic drugs including antipsychotics, antidepressants, and mood stabilizers in BD has been associated with an increased risk of stroke.^35^ Studies have also demonstrated that BD patients are prone to inflammation and endothelial dysfunction.^36^ Changes in circulating inflammatory and anti-inflammatory cytokines have been found in BD patients.^37^

Higher levels of circulating inflammatory cytokines causes endothelial cells to release vascular cell adhesion molecules which in turn recruit monocytes and T lymphocytes to initiate the atherosclerotic. Atherosclerosis is an important precursor to stroke.^38^ Endothelial dysfunction in BD leads to reduced vasoactive, anticoagulant, and anti-inflammatory status resulting due to altered availability of nitric oxide in the endothelium. This increases the risk of several cardiovascular disorders like hypertension, hypercholesterolemia, obesity, and diabetes.^39^ Such endothelial dysfunction is known to persists even with pharmacological therapy in BD patients and hence constitutes a trait marker of such patients causing increase risk of adverse cardiovascular events like stroke.^39^ Also, the presence of a hypercoagulable state in BD patients due to platelet hyperactivity alters the hemostatic function and heightens the risk of stroke.^39^-^41^ Platelet aggregation has been linked with serotonin metabolism, which is perturbed both in stroke patients as well as in BD.^42^,^43^

### Limitations

The current review should be interpreted with some limitations. There were just eight studies in the review. Despite the large sample size, data was predominantly available from selected countries with a robust health record system and hence the association may not be reflective of the global population. Secondly, the confounders were not similar across the studies. Many studies missed important cardiovascular risk factors like hyperlipidaemia, atrial fibrillation, smoking, obesity, sedentary lifestyle, etc. in the multivariate analysis. Identification of BD and stroke was also mostly by medical records in the included studies. It is plausible that errors in data record keeping may have induced bias. Studies did not differentiate between stroke types hence it is unclear if there is difference in the risk of ischemic and hemorrhagic stroke. Furthermore, it is unclear if treatment for BD affects the risk of stroke. None of the studies presented data on psychotherapy, counselling intervention, and drug therapy and its effects on the risk of stroke.

Nevertheless, the results of the review have important clinical implications. BD patients should be closely monitored for thromboembolic events and stroke symptoms to provide early and comprehensive care. Caregivers should be sensitized regarding such increased risk so that appropriate lifestyle modifications may be incorporated.

## CONCLUSIONS

Patients with BD may have an increased risk of stroke in comparison to the general population. The results are limited by the retrospective nature of the data and high inter-study heterogeneity which demand caution in the interpretation of the outcomes.

### Authors’ contributions:

**LG:** Conceived, designed the study and prepared the manuscript.

**FW** and **JY:** Collected the data and performed the analysis.

All authors have read, approved the final manuscript and are responsible for the integrity of the study
